# VibroIntegração BioMolecular: a structured multimodal protocol for autonomic regulation in community healthcare

**DOI:** 10.3389/fpubh.2026.1770221

**Published:** 2026-05-29

**Authors:** Evadison Soares Braga, Fernanda de Souza Teixeira

**Affiliations:** 1 NEUROPHYS Research Group, Federal University of Pelotas (UFPel), Pelotas, Rio Grande do Sul, Brazil; 2 NEUROPHYS Research Group, Superior School of Physical Education and Physiotherapy, Federal University of Pelotas, Pelotas, Rio Grande do Sul, Brazil

**Keywords:** autonomic regulation, community health, integrative and complementary practices, psychophysiological coherence, soft health technologies, VibroIntegração BioMolecular

## Abstract

VibroIntegração BioMolecular® (VIB) is a structured, low-cost multimodal protocol designed to support autonomic regulation in community healthcare settings. Organized into four sequential stages—guided muscle relaxation, resonance frequency breathing, multisensory interoceptive integration, and psychoeducation in a regulated state—the protocol aims to facilitate cardiorespiratory synchronization and self-regulation skills. This descriptive case series describes the implementation of VIB in primary care and community-based programs in southern Brazil (*n =* 38; 21 in primary care and 17 in palliative care settings). Physiological monitoring included heart rate variability (HRV)-based coherence monitoring, heart rate trends, respiratory rate, and subjective markers assessed longitudinally across sessions. Representative intra-individual changes included an increase in high-coherence from 0 to 53% and a reduction in low-coherence from 80 to 16% in a cardiovascular case. Similar directional patterns were observed across the four monitored cases. No adverse events were recorded, and the protocol was implemented without modification of prescribed medical treatments. While the study design does not allow causal inference, the findings suggest that the structured sequential organization of relaxation and resonance breathing may facilitate short-term autonomic modulation in real-world community environments. These preliminary results support the feasibility, safety, and reproducibility of the VIB protocol and provide a foundation for future controlled investigations.

## 1 Introduction

Traditional, Complementary and Integrative Medicine (TCIM) plays a significant role in global health, being used by a large portion of the world population as a primary or complementary form of care (1). The WHO Gujarat Declaration (2) reinforces the need for integrative practices that are safe, accessible, and supported by evidence. In Brazil, the National Policy on Integrative and Complementary Practices (PNPIC) structures and legitimizes these approaches within public services, promoting user-centered and culturally sensitive strategies of care. VibroIntegração BioMolecular® (VIB) emerges within this context as a low-cost multimodal intervention that integrates guided muscle relaxation, resonance breathing, multisensory stimulation, and psychoeducation delivered in a regulated physiological state. The sequential logic of the protocol—relaxation → breathing → multisensory modulation → psychoeducation—is designed to facilitate autonomic regulation, a construct supported by established models of neurovisceral integration and heart rate variability research (3–5). The protocol may support improvements in interoception and learning processes under conditions of reduced sympathetic activation (6, 7), and may contribute to reduction of allostatic load through structured autonomic pacing. These proposed mechanisms remain theoretical and require longitudinal validation. This structured sequence distinguishes VIB from techniques applied in isolation and may help explain the therapeutic observations reported in community settings.

## 2 Community context and CASE scenario

The VIB protocol has been applied over the past 2 years in real-world community healthcare settings in southern Brazil, including the Areal Leste Primary Health Care Unit (Pelotas, RS), the Cuidativa/UFPel Regional Reference Center for Palliative Care, and the Dharma Institute, an integrative health initiative. These services operate within the framework of the Brazilian National Policy on Integrative and Complementary Practices (PNPIC) and provide free or low-cost assistance to socially vulnerable populations.

At the Areal Leste Primary Health Care Unit, 21 individuals initiated participation in the protocol, of whom 6 discontinued follow-up due to personal or logistical reasons unrelated to adverse effects. At the Cuidativa center, 17 participants began the intervention, and 5 discontinued participation during the follow-up period. Sessions were conducted individually or in small groups ranging from 5 to 12 participants, depending on service capacity and demand.

Participants presented heterogeneous clinical conditions, including chronic stress, mild respiratory dysfunction, cardiovascular conditions, oncology patients in stable clinical follow-up, metabolic disorders, and emotional regulation difficulties. No conventional medical treatments were modified during participation in the VIB protocol, which was applied exclusively as an adjunct integrative practice.

The intervention was structured to be operationally accessible within primary care environments. It requires no specialized medical equipment, minimal infrastructure, and is delivered through weekly sessions lasting approximately 45 to 60 min. After supervised instruction, participants were encouraged to perform structured home-based self-application involving guided muscle relaxation (contraction–release sequences), resonance breathing practice, and brief interoceptive grounding exercises. This home-based continuation reduces long-term dependency on supervised sessions and contributes to economic feasibility in community settings. Adherence to home practice was self-reported.

To illustrate applicability and feasibility, representative observational cases are presented in Section 8.1. Across settings, observed outcomes included progressive redistribution toward HRV-based resonance patterns, reduction in time spent in non-resonant respiratory states, improved subjective respiratory comfort, emotional stabilization, and increased engagement in self-care behaviors. These findings derive from an observational case series and do not establish causal inference; however, they support the feasibility, acceptability, and operational sustainability of the protocol within public health contexts.

From the total sample (*n =* 38), four participants with complete and consistent longitudinal HRV monitoring were selected for detailed illustrative analysis. Selection criteria included completeness and continuity of longitudinal data, consistency of monitoring conditions across sessions, and availability of comprehensive clinical documentation across different clinical contexts.

These cases were selected to illustrate intra-individual longitudinal patterns under standardized observational conditions. It is acknowledged that these selected cases may not represent the full variability of the participant group, and selection bias cannot be excluded.

## 3 Theoretical framework of the VIB sequential regulation model

The VibroIntegração BioMolecular® (VIB) protocol is grounded in established principles of autonomic physiology and respiratory regulation. Rather than proposing direct biomolecular effects, the protocol is structured around mechanisms that are physiologically measurable and supported by prior literature on autonomic modulation, neurovisceral integration, and cardiorespiratory interaction.

### 3.1 Muscle relaxation and autonomic modulation

Voluntary reduction of muscle tension has been associated with decreased afferent signaling from muscle spindles and reduced activation of stress-related reflex pathways. Psychophysiological research demonstrates that relaxation techniques may reduce sympathetic arousal and facilitate parasympathetic predominance through vagal modulation mechanisms.

Improved autonomic balance may indirectly influence peripheral perfusion via modulation of vascular tone. However, in the present study, these mechanisms are interpreted as theoretical physiological pathways rather than directly measured endpoints.

### 3.2 Resonance breathing and cardiorespiratory coupling

Slow-paced breathing within the resonance frequency range (approximately 0.1 Hz) has been widely documented to enhance heart rate variability (HRV) amplitude and promote baroreflex sensitivity (5, 8, 9, 12, 20, 23). This breathing pattern strengthens synchronization between respiratory and cardiovascular oscillations, leading to increased vagal tone and improved autonomic flexibility (3, 10).

In specific clinical contexts, such as stress-related hyperventilation patterns, paced breathing may contribute to stabilization of respiratory rhythm and improved gas exchange efficiency. In the present non-controlled descriptive study, no direct biochemical or blood gas measurements were performed; therefore, interpretations remain within the domain of autonomic regulation and HRV dynamics.

### 3.3 Sensory integration and emotional regulation

The multisensory phase of the VIB protocol incorporates controlled low-intensity stimuli designed to support interoceptive awareness. Evidence from affective neuroscience suggests that interoceptive processing is linked to limbic system engagement and emotional regulation (6, 7, 11, 13−15, 17, 19).

In the present study, no neuroimaging or neurotransmitter measurements were conducted. Accordingly, this phase is conceptualized as facilitating experiential integration under conditions of autonomic coherence rather than directly inducing measurable neurochemical changes.

### 3.4 Sequential integration hypothesis

The central hypothesis of the VIB model is that the structured sequence—muscle relaxation followed by resonance breathing and sensory integration—enhances the probability of achieving autonomic coherence as measured by heart rate variability patterns (3, 5, 18, 22). Rather than asserting direct biomolecular modulation, the present study evaluates measurable physiological indicators, specifically HRV coherence patterns, heart rate trends, and longitudinal clinical evolution, consistent with established models of neurovisceral integration (4). Future controlled trials will be necessary to investigate potential downstream molecular or neurochemical correlates.

### 3.5 Measured outcomes in the present study

The primary physiological outcome measured in this observational case series was HRV-based coherence monitoring during structured sessions. Secondary indicators included heart rate trends, respiratory rate, subjective respiratory perception, and longitudinal clinical observations. These outcomes were selected because they are non-invasive, reproducible, and consistent with established autonomic assessment frameworks. No molecular biomarkers, inflammatory markers, or neurochemical parameters were directly measured. Therefore, all interpretations are restricted to observable physiological modulation within supervised sessions.

**Figure 1:**
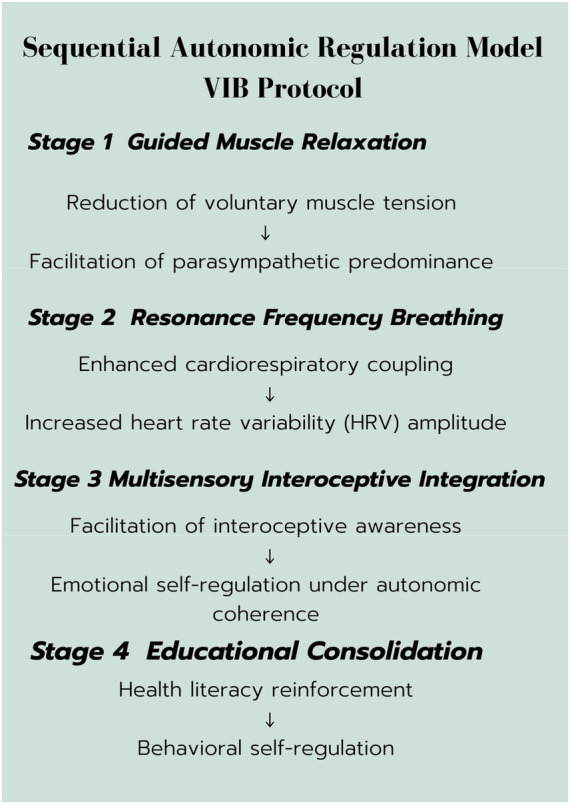
Sequential autonomic regulation model of the VibroIntegração BioMolecular® (VIB) protocol. The diagram represents the physiological progression of the method’s four stages: (1) Guided muscle relaxation; (2) resonance breathing; (3) multisensory stimulation; and (4) psychoeducation in a regulated state.

Figure 1 presents the conceptual organizational framework of the VIB protocol, illustrating the structured progression from voluntary muscle relaxation to resonance frequency breathing, followed by multisensory interoceptive integration and psychoeducational consolidation in a regulated physiological state. The model emphasizes measurable autonomic modulation, particularly heart rate variability (HRV)-based coherence patterns, as the primary observable outcome within supervised sessions. No direct molecular, inflammatory, or neurochemical parameters were assessed in the present study. Any potential downstream biological effects remain theoretical and require validation in controlled longitudinal investigations.

## 4 The VIB protocol (operation and sequence)

The VibroIntegração BioMolecular® (VIB) protocol is a structured, low-cost multimodal intervention organized into four sequential stages designed to facilitate autonomic regulation in community healthcare settings. Each stage prepares the physiological and attentional context for the next: guided muscle relaxation, resonance frequency breathing, multisensory interoceptive stimulation, and psychoeducation in a regulated state. The sequential organization aims to progressively reduce sympathetic predominance, facilitate cardiorespiratory synchronization, and support self-regulation skills. In the present observational study, physiological effects were evaluated primarily through HRV-based coherence monitoring.

### 4.1 Frequency and duration of sessions

The VIB protocol is applied in weekly sessions lasting approximately 45–60 min. This frequency was adopted in community settings to allow gradual familiarization with autonomic regulation practices. The structured repetition of sessions aims to support progressive respiratory stabilization, increased interoceptive awareness, improved self-perception of stress patterns, and reinforcement of self-regulation strategies.

Participants are encouraged to perform home-based breathing exercises and brief relaxation micro-sequences between sessions. Adherence is self-reported and not externally controlled.

### 4.2 Stage 1—guided muscle relaxation (5–10 min)

The first stage consists of progressive contraction–release sequences of major muscle groups, gentle guided movements, and proprioceptive scanning. The goal of this stage is voluntary reduction of global muscular tension and increased body awareness. In psychophysiological literature, muscle relaxation techniques are associated with decreased perceived stress and potential reduction in sympathetic arousal. In this study, no direct measurement of vascular or metabolic parameters was performed during this phase. Its role within the protocol is preparatory, creating conditions for subsequent paced breathing practice.

### 4.3 Stage 2—resonance frequency breathing (15–20 min)

After muscular tension is voluntarily reduced, participants engage in paced breathing within the resonance frequency range (approximately 5–6 cycles per minute). Emphasis is placed on diaphragmatic expansion, slow nasal breathing, rhythm stabilization, and gentle synchronization between breath and cardiac perception. Resonance breathing is widely documented to increase heart rate variability (HRV) amplitude and enhance baroreflex sensitivity. In the present study, this stage was the primary contributor to measurable HRV-based coherence redistribution.

No biochemical, gasometric, or nitric oxide measurements were performed; therefore, interpretations remain within the domain of autonomic modulation as assessed by HRV dynamics.

### 4.4 Stage 3—multisensory interoceptive stimulation (10–15 min)

Once cardiorespiratory coherence is established, low-intensity sensory stimuli may be introduced, including light tactile stimulation, controlled aromatic exposure, low-frequency auditory inputs, guided imagery, and grounding techniques. The purpose of this stage is to facilitate interoceptive awareness under conditions of reduced autonomic reactivity. Although literature suggests limbic involvement in affective touch and olfactory modulation, no neurochemical or neuroimaging assessments were performed in this study. Thus, effects are interpreted as experiential and behavioral rather than molecular.

### 4.5 Stage 4—psychoeducation in a regulated state (10–15 min)

Psychoeducation is delivered after the participant demonstrates observable respiratory stabilization and subjective reports of safety or calm. This stage includes explanation of bodily responses to stress, identification of thoracic versus diaphragmatic breathing patterns, practical instruction for daily application, and reinforcement of self-regulation strategies. Learning under reduced sympathetic activation may facilitate cognitive integration; however, neural plasticity was not directly measured in this study. Participants receive individualized guidance for home practice.

### 4.6 Core operational principles

The VIB protocol is structured around three operational principles. First, sequence organization may influence autonomic engagement: relaxation → breathing → sensory integration → psychoeducation. Second, autonomic stabilization precedes cognitive reframing, as reduced physiological reactivity may increase receptivity to health education. Third, low-cost body-based techniques can be implemented in community environments. These principles support feasibility in primary care and community-based contexts. In this study, effectiveness was evaluated descriptively through HRV-based coherence monitoring rather than controlled clinical endpoints.

## 5 Data collection and documentation

Data collection followed a structured observational framework designed to document physiological and subjective changes during participation in the VIB protocol. Documentation procedures aimed to ensure consistency of records across sessions while preserving the real-world character of the community-based intervention.

Before each session, baseline physiological parameters were recorded, including respiratory rate (manual 60-s count or digital monitoring), heart rate (digital oximeter or heart rate monitor), oxygen saturation (digital oximeter), and blood pressure (calibrated digital or aneroid sphygmomanometer). Respiratory pattern was qualitatively observed with attention to thoracic versus diaphragmatic movement predominance. Participants also reported subjective perceptions using simple analog scales for stress, anxiety, fatigue, and pain.

During the session, therapists documented qualitative observations related to muscular tension release, breathing rhythm stabilization, and participant-reported emotional responses. When available, HRV-based respiratory coherence monitoring was recorded using standardized digital tools (CardioEmotion®). It is important to clarify that the term “coherence” used in this study refers to a device-based operational classification provided by the CardioEmotion® system and does not correspond to standard HRV metrics as defined by international guidelines (5).

In this context, coherence reflects patterns of cardiorespiratory synchronization associated with respiratory sinus arrhythmia and baroreflex-mediated oscillations, commonly described in the literature as cardiovascular resonance phenomena (10).

While such synchronization has been associated with adaptive physiological regulation, the coherence metric used in this study should not be interpreted as a direct or quantitative measure of autonomic balance. Instead, it represents an operational descriptor of synchronization patterns observed during guided breathing sessions.

HRV-based coherence levels (high, intermediate, and low) were derived from device-based classification of cardiorespiratory synchronization patterns using the CardioEmotion® biofeedback system. These categories reflect the temporal distribution of physiological coherence states during guided sessions rather than spectral HRV analysis. Monitoring was used as a descriptive and instructional tool to document intra-session autonomic modulation, and redistribution among resonant, intermediate, and non-resonant breathing patterns was documented longitudinally.

After each session, post-intervention respiratory rate, perceived ease of breathing, emotional state, and relevant functional changes (sleep quality, mobility, pain perception) were recorded. Adherence to the home-based breathing and relaxation protocol was self-reported.

Longitudinal documentation allowed descriptive comparison of pre- and post-session trends and progressive redistribution of respiratory coherence patterns across sessions. In selected cardiopathic, oncological, and metabolic cases, independent clinical examination records were included descriptively when available; however, no laboratory or imaging outcomes were prospectively controlled within the study design.

Interoceptive perception was documented through structured qualitative self-report and therapist-guided reflective inquiry rather than validated psychometric instruments. Therefore, changes in interoceptive awareness are described descriptively and should not be interpreted as quantified psychometric outcomes.

All data were recorded either digitally or in standardized physical forms and stored in password-protected systems or secure physical archives. Access was restricted to responsible therapists and technical supervisors when necessary. For research purposes, all data were anonymized prior to analysis.

Operational ethical procedures followed principles of confidentiality, informed consent, and non-interference with ongoing medical treatments. The present study represents an observational case series and does not involve experimental manipulation or modification of conventional medical care.

## 6 Training, self-application, and safety

The VibroIntegração BioMolecular (VIB) protocol was designed for structured implementation in community and clinical settings, with emphasis on operational safety, accessibility, and feasibility. The protocol may be delivered by trained professionals and, after appropriate instruction, may also be performed in a supervised self-application format. This operational flexibility represents a distinct characteristic of the method, expanding its applicability in community, home-based, and health-promotion contexts.

Training for self-application includes foundational instruction in resonance breathing, recognition of individual basal respiratory rhythm, simple progressive muscle contraction–relaxation techniques, and identification of signs of comfort, physiological regulation, and autonomic overload. Participants are instructed in structured home micro-sequences to support continuity of practice between supervised sessions. This modality encourages autonomy and long-term engagement while maintaining principles of physiological safety.

Professionals applying the VIB protocol in clinical or community environments are expected to demonstrate competence in cardiorespiratory physiology and heart rate variability (HRV), trauma-informed communication, guided somatic relaxation techniques, and recognition of autonomic dysregulation signs. Structured training supports consistent application of sequential stages and safe adaptation for individuals presenting diverse clinical conditions. This preparation aims to maintain procedural coherence rather than to establish experimental reproducibility.

Safety principles are central to implementation. Prolonged breath retention is avoided, particularly in individuals with respiratory vulnerability or anxiety. Sensory stimuli intensity is adapted according to individual tolerance. Practice is interrupted in the presence of dizziness, acute discomfort, or signs of sympathetic hyperactivation. Emotional limits are respected at all times, and a calm, non-invasive environment is maintained. Participants presenting warning signs or persistent symptoms are referred for appropriate medical evaluation.

The operational accessibility of the VIB protocol derives from its reliance on low-cost body-based techniques, minimal infrastructure requirements, and structured self-application following supervised instruction. These characteristics make the protocol suitable for community programs, low-income populations, guided home-based practice, health education initiatives, and complementary use alongside conventional medical treatments. Its conceptual framework is grounded in current literature on autonomic regulation, interoception, and regulated-state learning; however, therapeutic efficacy requires validation through controlled longitudinal research.

## 7 Discussion

This observational case series aimed to describe physiological patterns observed during the implementation of the VibroIntegração BioMolecular® (VIB) protocol in a community healthcare setting. The primary measurable outcome was heart rate variability (HRV)-based coherence monitoring during structured sessions.

Across participants, a progressive intra-individual redistribution toward higher coherence states was observed. In the cardiovascular case, high coherence increased from 0 to 53%, while low coherence decreased from 80 to 16% over monitored sessions. Similar directional trends were observed in other participants, with gradual increases in green-zone distribution and corresponding reductions in red-zone dominance.

These findings suggest that the sequential structure of guided relaxation followed by resonance frequency breathing may facilitate short-term autonomic modulation, as reflected by HRV coherence patterns (3, 9). The consistency of intra-session improvements supports the hypothesis that structured pacing and voluntary muscle relaxation may enhance cardiorespiratory synchronization, consistent with models of neurovisceral integration (4).

Although HRV coherence was monitored using the CardioEmotion® biofeedback device during supervised sessions, the core intervention relies on slow, diaphragmatic, physiologically grounded breathing patterns that can be performed without continuous digital monitoring. Biofeedback was used as an instructional and observational tool to document autonomic modulation; however, the respiratory component of the protocol is inherently self-applicable once participants are properly trained. This characteristic contributes to the accessibility and feasibility of implementation in community contexts.

Importantly, this study does not claim direct biomolecular or neurochemical modulation. No molecular markers, neurotransmitter levels, or inflammatory biomarkers were directly measured. Observed clinical laboratory improvements during follow-up occurred in parallel with the intervention but cannot be attributed causally to the protocol.

From a safety perspective, no adverse events were recorded during implementation in either primary care or palliative care contexts. The intervention required minimal equipment, was low-cost, and was applied without modification of prescribed medical treatments. This supports the feasibility of implementing structured autonomic regulation protocols in community healthcare environments, including formats that allow supervised self-application after initial instruction.

The sequential nature of the VIB protocol may represent an organizational framework rather than a novel physiological mechanism. Its potential contribution lies in the structured integration of relaxation, paced breathing, and interoceptive awareness within a format adaptable to community-based care.

Given the observational design, results must be interpreted cautiously. The absence of a control group prevents causal inference. Improvements in HRV coherence may reflect learning effects, increased familiarity with the biofeedback device, voluntary breathing control, or nonspecific relaxation responses, phenomena commonly described in HRV biofeedback research (3). Importantly, the observed clinical and physiological improvements cannot be directly attributed to the VIB protocol. Multiple external factors, including ongoing medical treatments, lifestyle changes, spontaneous variation, and nonspecific effects related to attention and relaxation, may have contributed to the observed outcomes.

The VIB protocol is structured based on the theoretical assumption that the sequential organization of its components—muscle relaxation, resonance breathing, multisensory stimulation, and psychoeducation—may influence physiological receptivity and self-regulatory processes. However, this proposed sequencing effect remains a conceptual framework and was not directly tested within the present observational study.

Therefore, the findings should be interpreted as descriptive and hypothesis-generating, supporting the need for future controlled studies to investigate the potential role of intervention sequencing in autonomic regulation and health-related outcomes.

Future studies should include controlled designs, larger samples, validated autonomic metrics, and longer follow-up periods to determine whether observed short-term coherence modulation translates into sustained clinical outcomes.

### 7.1 Observational results: HRV coherence monitoring

The results presented in this section include different levels of evidence: (i) device-based HRV coherence data obtained during supervised sessions, (ii) patient-reported subjective outcomes, and (iii) independently obtained clinical data (e.g., laboratory and imaging findings). These categories should be interpreted separately, as they reflect distinct observational domains and were not integrated through controlled analytical methods.

Heart rate variability (HRV)-based coherence monitoring was performed during structured implementation of the VibroIntegração BioMolecular® (VIB) protocol in participants presenting distinct clinical comorbidities. Three standardized time points (initial, intermediate, and final session) were selected for comparative intra-individual analysis.

Percentages represent time spent in high (green), intermediate (blue), and low (red) coherence ranges during monitored sessions. Findings reflect short-term autonomic modulation observed under guided resonance breathing conditions. Given the observational design and absence of a control group, results are descriptive and do not imply causal inference.

In the cardiovascular case (Lily), high coherence increased from 0% at baseline (17/05) to 53% in the final recorded session (13/06), while low coherence decreased from 80 to 16%. Intermediate coherence showed relative stabilization over time. Similar directional patterns were observed among the monitored cases, with gradual increases in high-coherence distribution and corresponding reductions in low-coherence dominance. These changes were observed during structured sessions and reflect short-term autonomic modulation under guided resonance breathing conditions.

Table 1 presents longitudinal HRV-based coherence distribution for four participants monitored during implementation of the VibroIntegração BioMolecular® protocol. Percentages reflect time spent in high (green), intermediate (blue), and low (red) coherence ranges during each recorded session. Data represent intra-individual progression and do not imply causal inference.

**Table 1:** Longitudinal HRV-based coherence distribution across participants.

Cardiovascular case – Lily (chronic cardiovascular condition)			
Session	High (%)	Intermediate (%)	Low (%)
Initial (17/05)	0	20	80
Intermediate (20/05)	15	35	50
Final (13/06)	53	31	16

Oncological case – Rose (oncological follow-up)			
Session	High (%)	Intermediate (%)	Low (%)
Initial	12	38	50
Intermediate	25	42	33
Final	41	37	22

Oncological case – Magnolia (oncological follow-up)			
Session	High (%)	Intermediate (%)	Low (%)
Initial	8	29	63
Intermediate	19	41	40
Final	36	39	25

Glycemic dysregulation case – Daisy (type 2 diabetes mellitus)			
Session	High (%)	Intermediate (%)	Low (%)
Initial	14	33	53
Intermediate	28	36	36
Final	44	35	21

HRV-based coherence monitoring across four participants during implementation of the VibroIntegração BioMolecular® (VIB) protocol. Percentages represent time spent in high, intermediate, and low coherence ranges during structured sessions.

No inferential statistical analysis was performed due to the small heterogeneous sample and observational case-series design. HRV-based coherence changes are presented descriptively to illustrate intra-individual modulation during supervised sessions.

#### 7.1.1 Structured clinical evolution – cardiopathic case (Lily)

A consolidated longitudinal description across 10 sessions documented progressive symptom modulation. Initial sessions were characterized by chest discomfort and dizziness, followed by interventions including resonance breathing, guided meditation, reflexology, and essential oil application. Over successive sessions, reports included calmer breathing and reduction of dizziness, improvement in sleep pattern, consolidation of respiratory rhythm, reduction of muscular tension, improved digestion, and decreased perceived stress.

Session-by-session observations included reduction in sinus pressure, digestive discomfort, fatigue, emotional overload, leg numbness, irritability, cervical tension, and joint discomfort. In later sessions, participants reported subjective sensations described as “reset-like” experiences, marked reduction of cervical discomfort, perceived improvement in digestive function, subjective ease of breathing, and emotional stabilization.

Domain comparison before and after the observational period indicated changes from frequent chest discomfort to rare or absent episodes; short and irregular breathing to deep and coherent respiratory rhythm; irregular sleep to deep and continuous sleep; high stress perception to lower perceived stress; high muscular tension to reduced neuromuscular tension; and limited functionality to improved functional capacity. These descriptions reflect structured clinical documentation and do not represent controlled clinical endpoints.

#### 7.1.2 Clinical evolution summary – oncological case (Rose)

Independent clinical monitoring during the observational period included bone scintigraphy, computed tomography findings, and inflammatory laboratory parameters. Prior documentation described diffuse and intense metabolic activity in multiple sites, osteolytic lesions, adrenal nodular findings, fatigue, pain, emotional stress, poor sleep, and reduced vitality.

During the period in which the VIB protocol was implemented as an adjunct integrative practice, clinical follow-up indicated localized and reduced uptake on scintigraphy, radiological stabilization without evidence of new lesions, fluctuation and subsequent reduction in certain inflammatory laboratory parameters, controlled or absent pain, improved sleep depth, increased vitality, emotional stabilization, and improved adherence to daily self-care practices.

Independent clinical follow-up records indicated fluctuation and subsequent reduction in certain inflammatory laboratory parameters during the period in which the VIB protocol was applied as an adjunct integrative practice. These laboratory observations were derived from routine medical follow-up and were not prospectively controlled within the study design. Although no causal inference can be established, the temporal association warrants further controlled investigation into whether structured autonomic regulation sequences may influence systemic physiological markers under specific clinical conditions.

These findings were derived from independent medical follow-up and were not controlled within an experimental design. No modification of conventional oncological treatment occurred during the VIB implementation period.

#### 7.1.3 Clinical laboratory follow-up – Daisy

Independent laboratory monitoring demonstrated progressive reduction in glycated hemoglobin (HbA1c) levels during the period in which the VIB protocol was implemented as an adjunct integrative practice: 8.5% (03/07/2024), 8.1% (11/09/2024), 8.0% (06/01/2025), and 7.7% (28/01/2025). The estimated mean glucose at the last measurement was 174 mg/dL.

During the same observational period, the participant engaged in structured sessions including sequential muscle contraction–relaxation exercises, recovery of basal diaphragmatic breathing patterns (previously thoracic-dominant), and psychoeducation delivered under parasympathetic-dominant conditions.

It is important to emphasize that no causal relationship can be established between the VIB protocol and laboratory improvement. The present study is observational in nature and does not control for medication adjustment, dietary modification, lifestyle changes, or other medical variables.

However, the temporal association between autonomic regulation practices and clinical stabilization suggests that structured sequences involving muscle relaxation, respiratory recalibration, and regulated-state psychoeducation warrant further controlled investigation.

Similar autonomic coherence redistribution patterns were observed in the cardiovascular (Lily) and oncological cases (Rose and Magnolia), who also underwent the same sequential intervention structure.

Future longitudinal and controlled studies are required to determine whether structured autonomic sequencing may contribute to improved systemic regulation in populations with chronic conditions.

## 8 Conclusion

This observational case series documents the structured implementation of the VibroIntegração BioMolecular® (VIB) protocol in a Brazilian community healthcare setting and describes short-term autonomic patterns observed through heart rate variability (HRV)-based coherence monitoring.

The study positions VIB as an organizational framework that integrates guided muscle relaxation, paced resonance breathing, and regulated-state psychoeducation within primary and community care environments. The observed redistribution toward higher coherence states during supervised sessions suggests that sequential autonomic pacing may facilitate transient cardiorespiratory synchronization under guided conditions.

Findings derive from descriptive observation within a small and heterogeneous sample and must be interpreted in light of the inherent limitations of non-controlled designs. No biomolecular or neurochemical markers were directly measured, and no causal relationship is established between the intervention and observed clinical outcomes.

The principal contribution of this study lies in documenting the feasibility and practical applicability of a structured autonomic regulation sequence in real-world primary care and palliative care contexts without modification of conventional medical treatments. The protocol demonstrates that sequential integration of established practices—when applied coherently and within a physiologically regulated state—may represent a promising operational model for community-based health promotion.

Moreover, the observational findings suggest that integrated autonomic approaches may offer organizational advantages over fragmented application of isolated techniques, supporting further investigation into the role of muscular tension reduction, restoration of basal respiratory patterns, and structured autonomic modulation as central components in public health strategies.

Future studies employing controlled designs, larger samples, standardized autonomic metrics, and extended follow-up periods are required to determine whether short-term coherence modulation translates into sustained physiological or clinical outcomes.

## Data Availability

The original contributions presented in the study are included in the articles, further inquiries can be the corresponding authors.
